# TMC8 is associated with a paradoxical immune landscape and promotes clear cell renal cell carcinoma progression

**DOI:** 10.3389/fonc.2026.1979912

**Published:** 2026-10-30

**Authors:** Kuanyin Wang, Xingshu Zhu, Minghui Li, Lingling Lin, Na Lin, Lingfeng Zhu, Jin Chen

**Affiliations:** 1 Minimally Invasive Urology and Translational Medicine Center, Fuzhou First General Hospital Affiliated with Fujian Medical University, Fuzhou, Fujian, China; 2 Department of General Surgery, Lu’an Hospital Affiliated with Anhui Medical University, Lu’an, Anhui, China; 3 Institute of Clinical Medicine, The Second Affiliated Hospital of Hainan Medical University, Haikou, Hainan, China

**Keywords:** bioinformatics, ccRCC, immune infiltration, sexual dimorphism, TMC8, tumor microenvironment

## Abstract

**Background:**

Clear cell renal cell carcinoma (ccRCC) progression is closely linked to a complex tumor microenvironment (TME), yet key regulators remain unclear. This study aimed to systematically investigate the roles of *TMC8* in ccRCC, focusing on its immunological and biological potential.

**Methods:**

Multi-cohort transcriptomic data were integrated to analyze *TMC8* expression and prognostic value. Functional enrichment and multiple immune deconvolution algorithms were applied to characterize TME associations. Single-cell RNA sequencing (scRNA-seq) analysis characterized *TMC8*-associated transcriptional features across immune-cell populations. Sex-stratified analyses were performed to identify dimorphic effects. *In vitro* and *in vivo* experiments were conducted to validate the biological functions of *TMC8*.

**Results:**

*TMC8* was significantly upregulated in ccRCC and associated with poor overall survival, particularly in males. Its expression correlated with higher estimated immune infiltration and antigen-presentation-related signatures, alongside elevated expression of exhaustion-associated markers. scRNA-seq analysis suggested the uncoupling of T-cell activation and effector competence. Notably, *TMC8* exhibited marked sex-specific associations, with stronger oncogenic and immunological associations in males. Functionally, *TMC8* promoted tumor progression by enhancing proliferation, migration, and invasion, which were suppressed upon *TMC8* knockdown *in vitro* and *in vivo*.

**Conclusion:**

*TMC8* is associated with dysregulated oncogenic signaling and an immunologically active yet dysfunctional TME in ccRCC, with pronounced sex-specific associations, and may represent a putative therapeutic target warranting further validation.

## 1 Introduction

Clear cell renal cell carcinoma (ccRCC), the predominant subtype of renal cell carcinoma (RCC), accounts for approximately 80% of RCC cases and remains a major cause of cancer-related mortality (1). Despite substantial advances in surgery, targeted therapy, and immune checkpoint inhibitors, clinical outcomes of ccRCC patients remain suboptimal (2). This is largely attributed to the intrinsic resistance of ccRCC to conventional chemotherapy and radiotherapy, together with its highly heterogeneous responses to immunotherapy, which is increasingly recognized to be shaped by a complex tumor microenvironment (TME) (3). Recent studies have shown that immune-related processes, including T cell activation, macrophage polarization, and immune checkpoint signaling, are closely involved in ccRCC progression and treatment response (3–5). Nevertheless, the molecular drivers coupling the immune microenvironment to tumor behavior remain unclear, particularly in the context of clinically relevant heterogeneity.

Transmembrane channel-like (TMC) proteins comprise a conserved family of membrane proteins that have been primarily studied in sensory biology, yet emerging evidence suggests their broader involvement in immune regulation and tumorigenesis (6–8). Among them, *TMC6* and *TMC8* are best characterized in the context of epidermodysplasia verruciformis (EV), where loss-of-function mutations disrupt the TMC6/TMC8/CIB1 complex, leading to impaired control of viral infection and increased susceptibility to cutaneous squamous cell carcinoma (6). Mechanistically, *TMC8* has been implicated in the regulation of intracellular zinc homeostasis and NF-κB signaling, as well as tumor necrosis factor receptor–associated pathways (7), suggesting a potential role in coordinating immune responses. Notably, recent studies indicate that the function of TMC6/TMC8 may extend beyond keratinocyte-intrinsic antiviral activity and involve broader CIB1-dependent mechanisms (6, 8). Despite these observations, the role of *TMC8* in solid tumors, particularly in shaping the tumor immune microenvironment, remains largely undefined.

Given the central contribution of immune regulation to ccRCC progression, we systematically investigated *TMC8* to provide new insights into the associations between TMC8 expression, tumor behavior, and TME characteristics. In this study, expression patterns and clinical relevance were first assessed across multiple independent cohorts, followed by functional enrichment analysis of *TMC8*-associated genes. Its relationship with TME, particularly immune infiltration and immune-related signatures, was then evaluated. Single-cell analysis further delineated the immune context of *TMC8*, alongside an exploration of sex-specific differences. Finally, both *in vitro* and *in vivo* experiments were conducted to validate its effects on tumor progression.

## 2 Materials and methods

### 2.1 Data collection and processing

A total of 1701 ccRCC samples were included in the work. The Cancer Genome Atlas (TCGA) log_2_(TPM + 0.001) data, International Cancer Genome Consortium (ICGC) data and Genotype-Tissue Expression (GTEx) data were downloaded from the University of California Santa Cruz (UCSC) platform (https://xena.ucsc.edu). The GSE53757, GSE40435, and GSE46969 profiles were retrieved from the Gene Expression Omnibus (GEO) database (https://www.ncbi.nlm.nih.gov/geo/). Representative immunohistochemistry (IHC) images regarding kidney cancer were obtained from the Human Protein Atlas (HPA) database (https://www.proteinatlas.org). The clinical characteristics of the datasets are summarized in **Supplementary Table 1**. The single-cell RNA sequencing (scRNA-seq) data originated from the GSE237429 dataset. The immune-related genes were acquired from the ImmPort database (https://www.immport.org/home), and the relevant transcriptomic factors were sourced from the Cistrome Project (www.cistrome.org). The R package “limma” was used to screen differentially expressed genes (DEGs) with the following parameters, an adjusted *p*-value<0.05 and |log_2_(fold change)|>1.

### 2.2 Correlation and survival analyses

The R packages “stats” and “rstatix” were used to perform Spearman’s rank correlation tests between *TMC8* expression and both transcription factors (TFs) expression and the proportion of tumor-infiltrating lymphocytes (TILs). Samples were stratified by the clinical characteristics, and difference analyses in *TMC8* expression as well as the infiltrating proportion of TILs across various clinical phenotypes were conducted. Patients were classified into high and low *TMC8* expression subgroups using the median expression value as a pre-specified cut-off. The R packages “survival” and “survminer” were used for Kaplan–Meier survival analysis and univariate (uniCox) and multivariable Cox proportional hazards analyses (multiCox) to assess associations with overall survival (OS) in ccRCC patients.

### 2.3 Function enrichment analysis and Tumor microenvironment estimation

The R package “clusterProfiler” and “org.Hs.eg.db” were applied for Gene Ontology (GO) and a Kyoto Encyclopedia of Genes and Genomes (KEGG) enrichment analyses. The single-sample Gene Set Enrichment Analysis (ssGSEA) was used to assess pathway-related and immune-cell-associated enrichment scores, based on the predefined gene sets in the Molecular Signatures Database (https://www.gsea-msigdb.org/gsea/msigdb). The 29 established immune signatures (9) were employed to investigate immune-related functions associated with TMC8 expression. The R package “estimate” was used to calculate Immune, Stromal, and ESTIMATE scores to assess the infiltration of immune and stromal cells. The R package “immunedeconv” was utilized to perform parallel analyses in immune infiltration using multiple algorithms, such as TIMER, CIBERSORT, xCell, ConsensusTME and others.

### 2.4 Single-cell RNA sequencing analysis

Single-cell RNA-seq data (GSE237429) were processed using Cell Ranger (v6.1.2, 10x Genomics) against the GRCh38 reference. Filtered matrices were analyzed via Seurat (v5). Quality control retained cells with 400–4, 000 detected genes, >1, 000 unique molecular identifiers (UMIs), and <20% mitochondrial reads. Doublets were removed using DoubletFinder (v2.0.3) with an 8% expected rate. Data were normalized via SCTransform (3, 000 variable features), regressing out mitochondrial and library size effects. Batch effects were corrected using Harmony (v0.1.1) on the principal component analysis (PCA) embeddings. Clustering was performed using the Louvain algorithm (resolution 0.1) on a shared nearest-neighbor graph, followed by t-distributed stochastic neighbor embedding (t-SNE) visualization. Major immune lineages were identified by canonical markers.

### 2.5 Cell culture and lentivirus transfection

The HK-2, Caki-2, A498, and 786-O cell lines were purchased from the Shanghai Institute of Cell Biology, Chinese Academy of Sciences in Shanghai, China. HK-2, Caki-2, and A498 cells were cultured in Dulbecco’s Modified Eagle Medium (DMEM) supplemented with 10% fetal bovine serum (FBS) and 1% penicillin/streptomycin (PS), while 786-O cells were cultured in RPMI-1640 medium under identical conditions. Cells were trypsinized with 0.25% trypsin, centrifuged, and passaged upon reaching 70-80% confluence. Three short hairpin RNA (shRNA) sequences targeting *TMC8* were designed (TMC8-sh1, 5’-GCTTCCTGCTGCTACTCAACC-3’; TMC8-sh2, 5’-CCTTCTACATCAAGAAGTACA-3’; TMC8-sh3, 5’-CCAAGTACTCACAGGACAACA-3’). Lentiviral vectors for *TMC8* knockdown were constructed by Hanbio Biotechnology (Shanghai, China). Cells were seeded in six-well plates, on the subsequent day, the medium was replaced with fresh medium containing 6 μg/mL polybrene (Yesen, Shanghai, China) prior to lentiviral suspension. Gene expression was evaluated after 48h cultivation.

### 2.6 Reverse transcription–quantitative PCR and western blotting

Total RNA was isolated from cells using TRIzol reagent (Vazyme, Nanjing, China). Reverse transcription was performed to synthesize cDNA using the HiScript II Q RT SuperMix for qPCR kit (Vazyme, Nanjing, China). Quantitative PCR (qPCR) was conducted using the ChamQ SYBR Color qPCR Master Mix kit (Vazyme, Nanjing, China). The primer sequences used for *TMC8* amplification are 5’-ACGGAGGTCAACCTCACTCTGA-3’ (forward) and 5’-GTTGTAGCCGTAGGACTCACAG-3’ (reverse). The primer sequences used for 18S amplification are 5’-CGACGACCCATTCGAACGTCT-3’ (forward) and 5’-CTCTCCGGAATCGAACCCTGA-3’ (reverse).

For western blotting, cells were lysed using RIPA buffer (Yeasen, Shanghai, China) containing protease inhibitors. Protein concentration was quantified using a BCA kit (Yeasen, Shanghai, China). Equal amounts of protein were resolved by SDS-PAGE, transferred onto PVDF membranes, and blocked with 5% non-fat milk. The membranes were incubated with primary antibodies (TMC8, Bioss, Beijing, China; β-actin, Proteintech, Wuhan, China) followed by HRP-conjugated secondary antibodies (Proteintech, Wuhan, China). Protein bands were visualized using ECL chemiluminescent reagent (Yeasen, Shanghai, China) and detected with a chemiluminescence imaging system. Band intensities were quantified using ImageJ software (version 1.8.0).

### 2.7 Cell proliferation assays

The Cell Counting Kit-8 (CCK-8) (Beyotime, Shanghai, China) and colony formation assay were used to examine cell proliferation. For CCK-8 assay, cells were seeded in 96-well plates at a density of 6, 000 cells/well (a volume of 100 μL per well) and incubated overnight. At 24, 48, and 72h, CCK-8 solution was added to each well to quantify the optical density of each well at 450 nm using a microplate reader. For the colony formation assay, 1, 000 cells per well were seeded in a 6-well plate and cultured for 14–21 days. The resulting colonies were fixed with 4% paraformaldehyde, stained with 0.1% crystal violet, and quantified using ImageJ software (version 1.8.0). For cell cycle analysis, cells were harvested and fixed in 75% ethanol at -20 °C. After fixation, cells were resuspended in phosphate-buffered saline (PBS) containing 0.2% Triton X-100 and 0.1% RNase A, and incubated at 37 °C for 30min. Finally, cells were stained with propidium iodide (PI), and then analyzed by flow cytometry to assess the cell cycle distribution. Cells were first gated by forward and side scatter to exclude debris, and doublets were excluded on the PI-area versus PI-width plot before cell-cycle analysis.

### 2.8 Cell migration assays

Wound healing and transwell assays were used to examine cell migration and invasion. Cells were incubated in a 6-well plate until they reached 100% confluence. The cell monolayer was scratched using a 200 µL pipette tip, rinsed with PBS, and then incubated in serum-free medium for 24h. Wound closure images were collected and analyzed to quantify the migration. Transwell membranes were enveloped with Matrigel (Yeasen, Shanghai, China). A total of 8×10^5^ cells/mL were seeded into the upper chamber in serum-free medium, while the lower chamber was filled with 600 μL of medium containing 15% FBS. After 48h incubation, cells that had invaded the lower surface were fixed with 4% paraformaldehyde, stained with 0.1% crystal violet, and photographed. Images of invading cells were captured and used to calculate cell invasive capacity.

### 2.9 Subcutaneous xenograft assay in nude mice

Female nude mice (4–6 weeks old) were purchased from GemPharmatech Co., Ltd. (Nanjing, Jiangsu, China) and housed under specific pathogen-free (SPF) conditions with a 12 h light/dark cycle and free access to food and water. For tumor cell implantation, mice were anesthetized by inhalation of isoflurane (RWD Life Science, Shenzhen, China) delivered via a calibrated vaporizer: induction at 3% isoflurane in 100% O_2_ in an induction chamber, followed by maintenance at 1.5–2% isoflurane via a nose cone during the procedure. Anesthetic depth was confirmed by the absence of pedal and corneal reflexes. A total of 12 mice were used (6 per group). Mice were randomly allocated to the shTMC8 and control groups, and tumor measurements were performed by an investigator blinded to group assignment. Logarithmic-phase cells (4×10^6^ cells per 100 μL, resuspended in serum-free medium) were subcutaneously injected into the right flank of each mouse using a 25-gauge needle. After implantation, mice were placed on a warming pad (37 °C) until full recovery of the righting reflex and then returned to their home cages. Tumor dimensions were measured at regular intervals with digital calipers to monitor growth kinetics. Body weight and signs of distress were monitored throughout the experiment. Once the tumors reached a predetermined endpoint, mice were euthanized by CO_2_ inhalation at a displacement rate of 30–70% of the chamber volume per minute, followed by cervical dislocation as a secondary physical method to confirm death, in accordance with the approved animal protocol. Subsequently, the xenograft tumors were excised, photographed, and weighed for further documentation.

### 2.10 Statistical analysis

Data are presented as the mean ± standard deviation (SD) of at least three independent experiments. R software (version 4.5.3) and GraphPad Prism 10.0 were used for statistical analysis. A log-rank test was used to assess statistical differences in the Kaplan–Meier analysis. Spearman’s rank correlation was applied for the correlation analysis. The 2^-△△Ct^ method was used to evaluate the results of qRT-PCR. ImageJ (version 1.8.0) and GraphPad Prism 10.0 were used to statistically calculate the cell mobility. Student’s t-test and one-way ANOVA were used to calculate the significant differences between groups. A *p*-value <0.05 was considered statistically significant. Differential expression analysis used Benjamini-Hochberg adjusted p-values; exploratory correlation and comparison analyses are reported with unadjusted p-values and are interpreted as hypothesis-generating.

## 3 Results

### 3.1 TMC8 presented high expression in ccRCC patients

*TMC8* expression in healthy human tissues was profiled using the GTEx database (**Supplementary Figure 1A**). Both TCGA (**Figure 1A**) and ICGC datasets (**Supplementary Figure 1B**) were utilized to conduct difference analysis in tumor versus normal tissues. Given the limited normal samples in TCGA cohort, GTEx data were integrated to achieve a more comprehensive comparison (**Supplementary Figure 1C**). Subsequently, the unpaired and paired difference analyses consistently suggested that *TMC8* expression was significantly elevated in kidney renal clear cell carcinoma (KIRC) patients (ccRCC) (**Figures 1A, B**). Furthermore, IHC staining from HPA database confirmed kidney cancer tissues exhibited higher TMC8 protein levels (**Figure 1C**). Higher *TMC8* expression was associated with worse overall survival (OS) in the overall cohort (*p* = 0.0463) and with a stronger association in male patients (*p* = 0.0079) (**Figures 1D, E**; **Supplementary Table 2**). A significant TMC8-by-sex interaction was observed for OS (*p*-value for interaction = 0.0394; **Supplementary Table 2**). *TMC8* expression was associated with OS in univariate but not multivariable Cox analysis (**Supplementary Table 3**).

**Figure 1:**
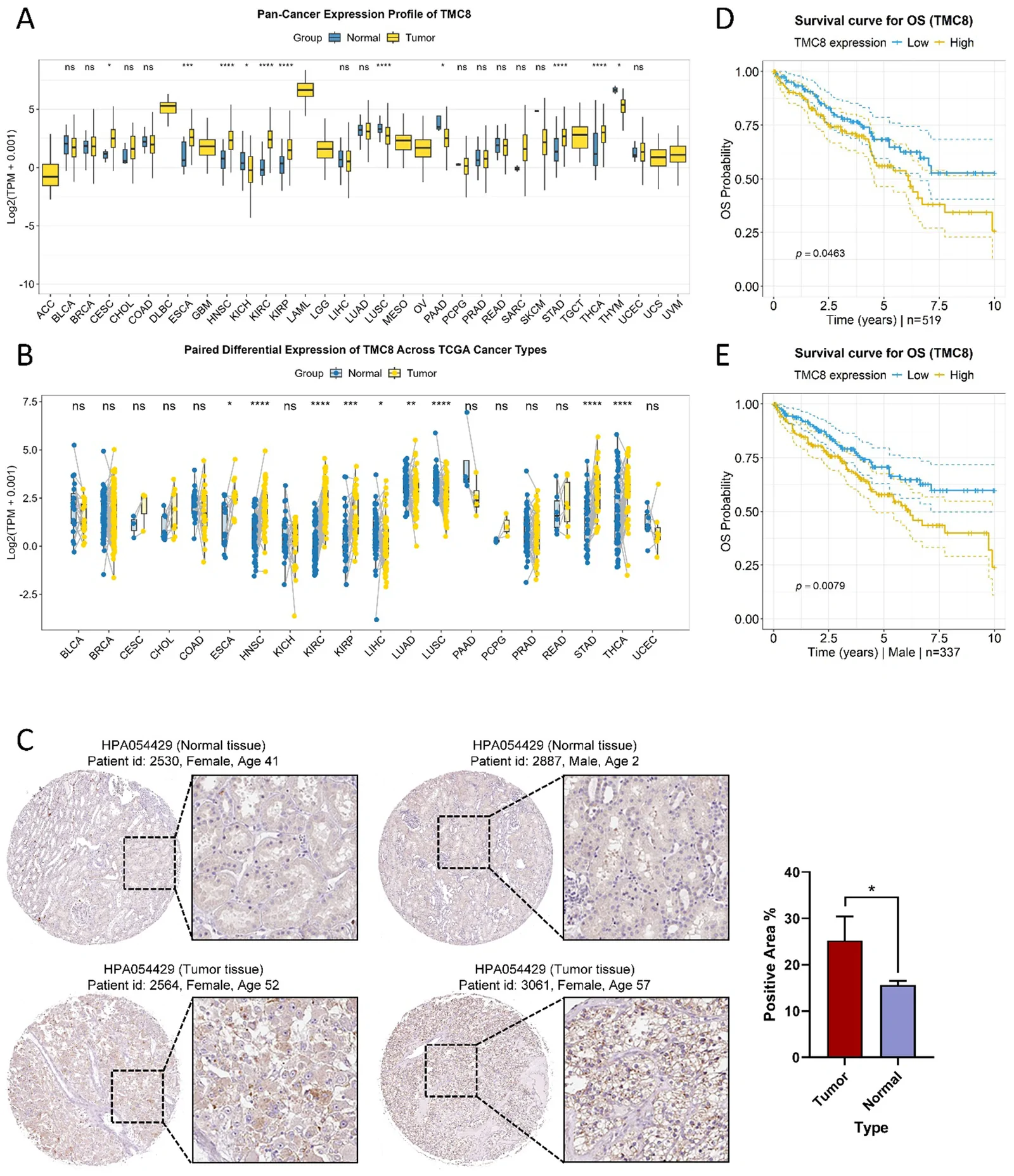
Expression profiles and prognostic significance of *TMC8* in ccRCC. **(A)** Elevated *TMC8* expression in ccRCC tissues compared to normal tissues in the TCGA cohort. **(B)** Paired difference analysis confirming higher *TMC8* expression in ccRCC versus adjacent normal tissues. **(C)** Representative immunohistochemistry (IHC) images from the HPA database showing high TMC8 protein expression in ccRCC tissues. **(D, E)** Kaplan-Meier survival curves indicating that high *TMC8* expression was associated with worse overall survival in the entire ccRCC cohort **(D)**, with a stronger association in male patients **(E)**. The ccRCC patients were classified into high and low TMC8 expression subgroups relative to the median TMC8 expression level. These survival analyses are exploratory and were performed without correction for multiple testing. *p < 0.05, **p < 0.01, ***p < 0.001, ****p < 0.0001.

Regarding clinicopathological characteristics, *TMC8* expression did not differ significantly across most clinical phenotypes (**Supplementary Figures 3A–D**), but it increased with advancing tumor stages and grades (**Supplementary Figures 3E, F**), and was highly expressed in the C2 (IFN-gamma Dominant), C3 (Inflammatory), and C6 (TGF-beta Dominant) immune subtypes (**Supplementary Figure 3G**). These findings implied that *TMC8* likely played critical roles in ccRCC tumorigenesis, mediated by shaping a complex tumor microenvironment.

### 3.2 TMC8 was involved in functions underlying ccRCC progression

To elucidate the roles of *TMC8* in ccRCC, we performed difference analysis and identified 2, 425 upregulated and 12 downregulated DEGs in the high *TMC8* expression subgroup versus the low subgroup (**Supplementary Figure 4A**). GO analysis revealed that these DEGs were significantly enriched in lymphocyte-mediated immunity, regulation of T cell activation, regulation of the immune effector process and other biological processes (**Supplementary Figure 4B**), a finding further supported by ssGSEA (**Supplementary Figure 4C**). In terms of cellular components, the DEGs were primarily involved in plasma membrane signaling receptor complex, T cell receptor complex, and blood microparticles. Additionally, molecular functions including antigen binding, immune receptor activity, and cytokine activity were identified (**Supplementary Table 4**). KEGG pathway analysis demonstrated that *TMC8* was associated with T cell receptor, chemokine, NF-kappa B, JAK-STAT, and Ras signaling pathways, as well as cytokine-cytokine receptor interactions (**Supplementary Figure 4D**; **Supplementary Table 5**). Collectively, these findings indicated that *TMC8* was not only associated with TME remodeling, but also with intrinsic oncogenic signaling axes known to promote cell proliferation and migration.

### 3.3 TMC8 was associated with formation of TME

TME scores (immune, stromal, and ESTIMATE scores) were significantly elevated in the tumor, advancing grades and the high *TMC8* expression tissues, as well as positively associated with *TMC8* expression levels (**Figure 2A**; **Supplementary Figures 5A–C**). Particularly, CD8+ T cells, macrophages, regulatory T cells (Tregs), cytolytic activity, immune checkpoints and other immune terms showed consistent results with TME scores in difference analysis and correlation test (**Figures 2B–D**; **Supplementary Figures 5D, E**; **Supplementary Table 6**). These correlations linked *TMC8* expression to immune-cell-associated signatures, particularly those related to T cells and macrophages. To assess consistency across computational methods, we estimated immune infiltration using multiple algorithms, revealing that CD4+ T cells, CD8+ T cells, and macrophages were significantly more abundant in ccRCC patients with high TMC8 expression (**Figure 2E**).

**Figure 2:**
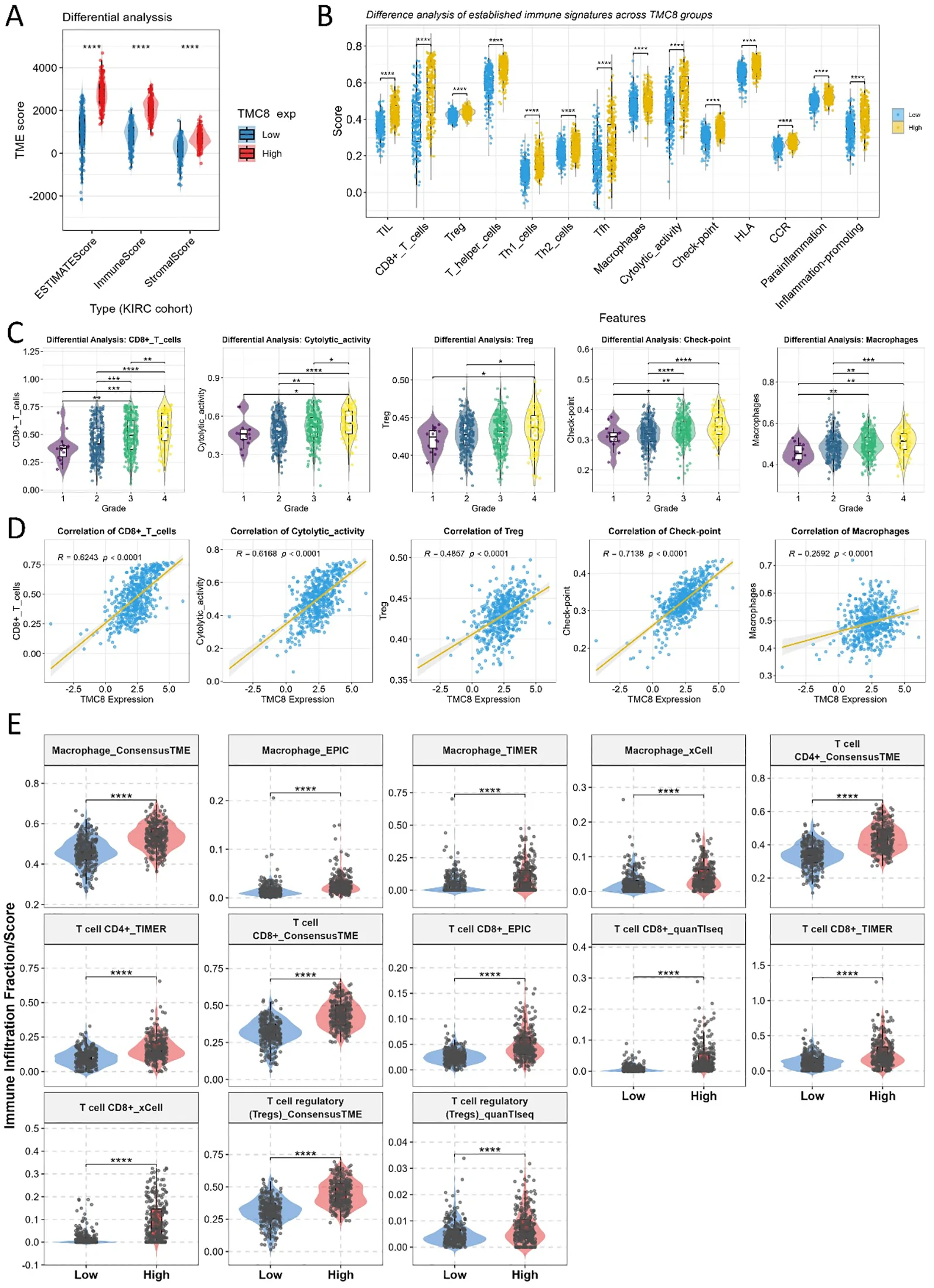
Associations between *TMC8* expression, TME scores, and estimated immune infiltration in ccRCC. **(A)** Difference analysis in TME scores between the ccRCC sample subgroups with high and low *TMC8* expression. **(B, C)** The proportions of TICs were significantly increased in the TMC8-high subgroup compared with the TMC8-low subgroup **(B)**, and in advanced-grade tumors compared with early-grade tumors **(C)**. **(D)** Correlation test of the proportion of tumor-infiltrating immune cells (TICs) with *TMC8* expression levels. **(E)** Macrophages, CD4+ and CD8+ T cells showing remarkably high infiltrating proportion in the high subgroup. *p<0.05, **p<0.01, ***p<0.001, ****p<0.0001.

### 3.4 TMC8-associated immune activation and concomitant exhaustion

To further characterize immune-related transcriptional features associated with *TMC8* expression, we profiled the expression patterns of 1, 417 immune-related genes and identified 406 DEGs (398 upregulated and 8 downregulated) between the two *TMC8* expression subgroups (**Figure 3A**). According to the predefined ImmPort pathways (**Supplementary Table 7**), T-cell receptor (TCR) signaling, immune checkpoint regulation, and T cell activation were exclusively populated by upregulated genes, indicating enrichment of immune activation-related transcripts (**Figure 3B**). Subsequently, 208 immune-related genes were demonstrated to be significantly associated with the poor OS (**Figure 3C**; **Supplementary Table 8**), and 87 common genes were shared by the prognostic markers and *TMC8*-associated DEGs (**Figure 3D**). Among the shared factors, the top five genes (*BATF*, *NCF4*, *NOD2*, *ZAP70*, and *ZBP1*) with the strongest positive correlation with *TMC8* expression were selected for further study (**Figure 3E**; **Supplementary Table 9**).

**Figure 3:**
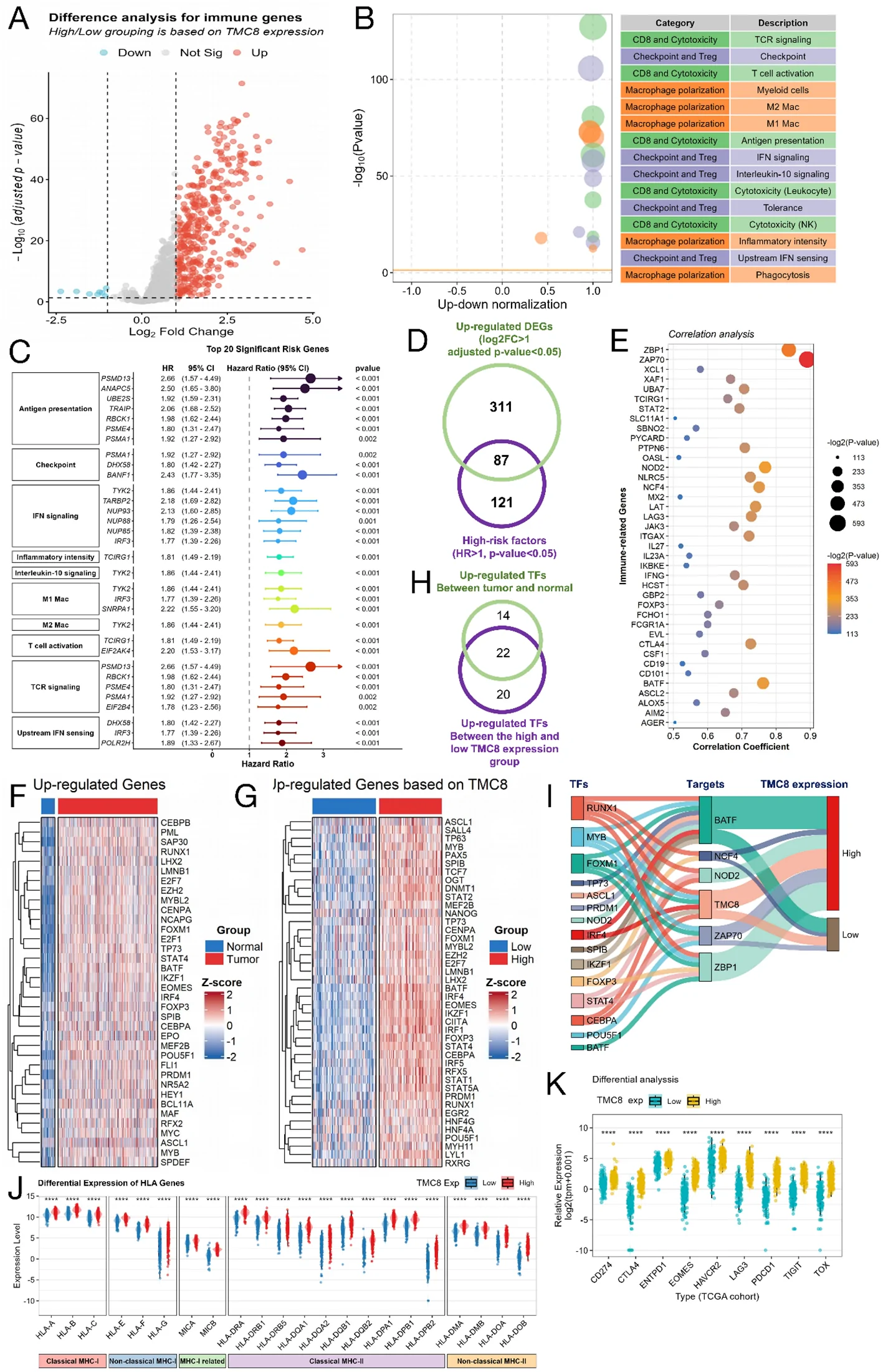
*TMC8*-associated immune-related genes and predicted upstream regulatory relationships. **(A)** Identification of differentially expressed genes (DEGs) among the immune-related genes (sourced from the ImmPort database) in the high TMC8 expression subgroup versus the low subgroup. An adjusted *p*-value <0.05 and |log_2_(fold change)| >1 were the cutoff criteria. **(B)** Functional categorization of the DEGs according to predefined ImmPort pathways. **(C)** Prognostic test on overall survival for the immune-related genes, presenting the top 20 significant high-risk factors. **(D)** Venn diagram identifying 87 overlapping genes shared by the difference analysis and prognostic test. **(E)** Correlation analysis of the overlapping factors, highlighting those with a correlation coefficient >0.5. **(F, G)** Difference analysis in transcription factors between ccRCC and normal tissues **(F)**, as well as between the ccRCC subgroups with high and low *TMC8* expression **(G)**. **(H)** Venn diagram showing 22 TFs shared by the two above difference analyses in **(F, G)**. **(I)** Sankey diagram illustrating predicted regulatory relationships based on OmniPath, ENCODE and ChEA databases. **(J, K)** Expression comparison of HLA-related genes **(J)** and exhaustion-related genes **(K)** between the two *TMC8* expression subgroups. ****p<0.0001.

Meanwhile, we retrieved 313 TFs from the Cistrome Project in order to identify candidate upstream regulators of *TMC8* and associated immune genes. Among these, 22 candidates were differentially expressed across both ccRCC tissues and the *TMC8* subgroups (**Figures 3F–H**; **Supplementary Table 10**). Based on target predictions from the ChEA, ENCODE, and OmniPath databases, we found *RUNX1*, *MYB*, *FOXM1*, *IRF4*, *IKZF1*, and *CEBPA* as candidate upstream regulators of *TMC8* (**Figure 3I**). Additionally, given the *TMC8*-associated difference in human leukocyte antigen (HLA) distribution (**Figure 2B**), representative *HLA* genes were broadly upregulated in the high TMC8 expression subgroup (**Figure 3J**), indicating higher expression of antigen-presentation-related genes. Alongside these antigen-presentation-related expression patterns, key T-cell exhaustion markers, including *PDCD1* (*PD1*), *LAG3*, *HAVCR2* (*TIM3*), *TIGIT*, and the master transcription factor TOX, were also significantly upregulated in the ccRCC patients highly expressing *TMC8* (**Figure 3K**). These findings indicate that high TMC8 expression is associated with concurrent elevation of antigen-presentation-related and exhaustion-associated transcripts.

### 3.5 The scRNA-seq analysis supported the uncoupling of T-cell activation and competence

Using canonical lineage markers, we successfully annotated CD8+ T cells, CD4+ T cells, macrophages and other immune cells within the TME (**Figures 4A–F**; **Supplementary Figure 6**). Within the subsets, *TMC8* expression was associated with distinct pathway enrichment patterns, including Th differentiation in CD4+ T cells, AMPK/Rap1 signaling in CD8+ T cells, and TNF/Apelin cascades in macrophages. Most strikingly, *TMC8* expression across T-cell populations revealed a synchronized enrichment for TCR signaling and antigen processing pathways (**Figure 4G**; **Supplementary Table 11**). Additionally, the cells with high *TMC8* expression exhibited significant upregulation of effector molecules (*GZMB*, *PRF1*, *IFNG*) alongside critical checkpoints (*PDCD1*, *LAG3*, *HAVCR2*) (**Figures 4H–M**). This dual enhancement is consistent with an uncoupling of T-cell activation and effector competence, offering a high-resolution molecular characterization of the dysfunctional immune microenvironment in ccRCC and corroborating our bulk transcriptomic findings at the single-cell level. Whether this concurrent enhancement reflects a causal relationship awaits functional validation.

**Figure 4:**
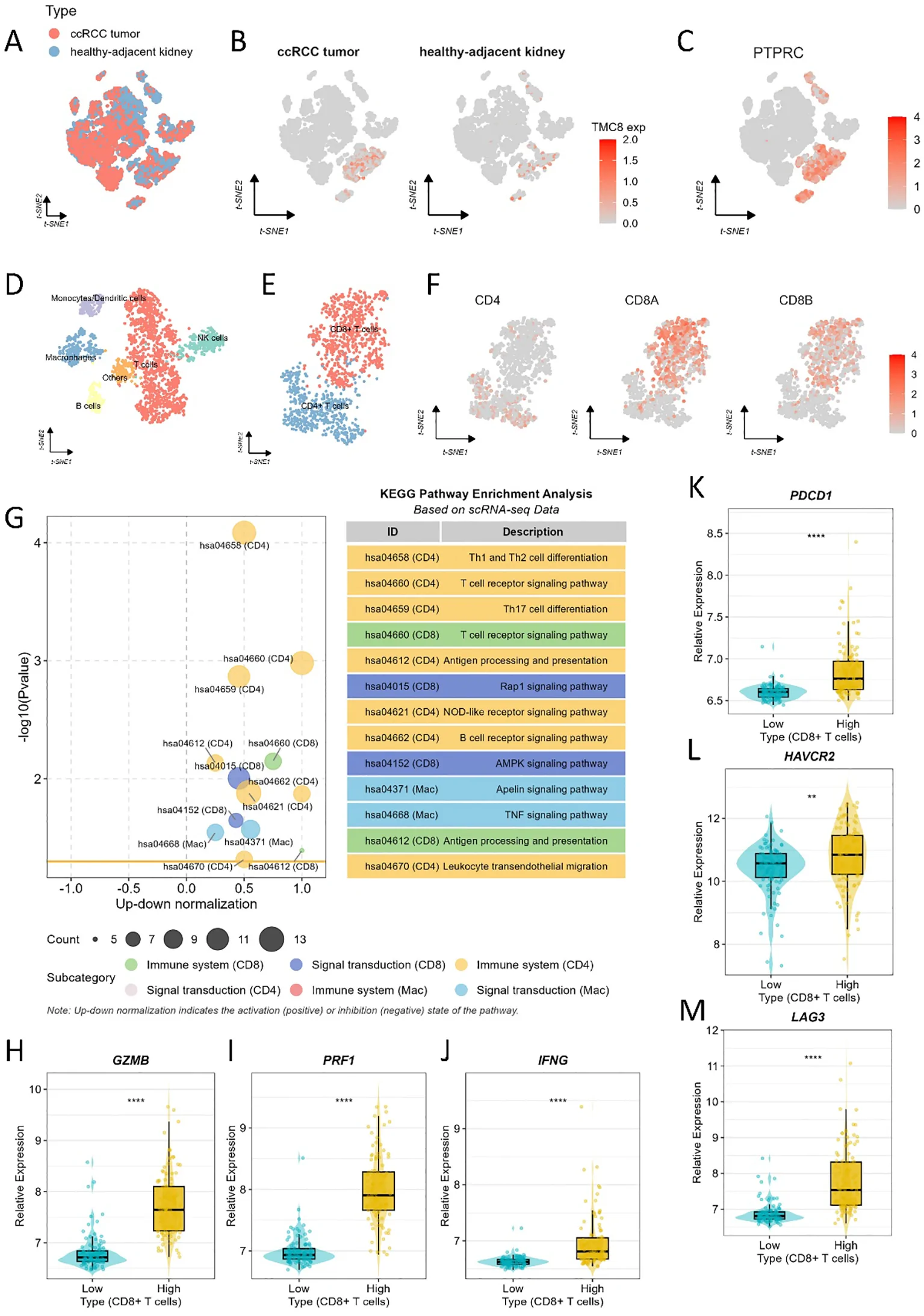
*TMC8*-associated immune landscapes characterized by scRNA-seq data. **(A–C)** *t-SNE* plots illustrating the tissue type distribution **(A)**, and *TMC8* **(B)** and *PTPRC* (CD45) **(C)** expression. **(D–F)** *t-SNE* visualizations of the immune cell types annotated on the basis of canonical lineage markers **(D)**, T cell subclusters **(E)**, and the expression of T cell markers (*CD4*, *CD8A*, and *CD8B*) **(F)**. **(G)** Bubble plot showing pathway enrichment associated with *TMC8* expression in macrophages, CD4+ T cells, and CD8+ T cells. **(H–M)** Boxplots comparing the expression levels of effector molecules (*GZMB*, *PRF1*, and *IFNG*) and immune checkpoints (*PDCD1*, *HAVCR2*, and *LAG3*) between TMC8-high and TMC8-low CD8+ T cells. **p<0.01, ****p<0.0001.

### 3.6 TMC8 shaped divergent TME between sexes

Although *TMC8* expression levels were comparable between the male and female patients (**Supplementary Figure 3A**), high *TMC8* expression was nominally associated with worse OS in males (**Figure 1E**), prompting further examination of sex-stratified associations. To illuminate this sex-stratified pattern, we firstly conducted difference analyses between the *TMC8* subgroups, and found that males exhibited more extensive differences in gene expression between the *TMC8* subgroups, characterized by 3, 633 upregulated and 101 downregulated male-specific DEGs (**Figures 5A–D**). Consequently, the *TMC8*-associated functional landscapes were divergent between the sexes (**Figure 5E**). In females, *TMC8* was preferentially associated with tissue remodeling and homeostasis, including homotypic cell-cell adhesion and the regulation of wound healing. Conversely, the male-specific network was heavily skewed toward mitotic aberrations, such as microtubule bundle formation and nuclear chromosome segregation, alongside robust chemokine responses (**Figures 5F, G**; **Supplementary Table 12**).

**Figure 5:**
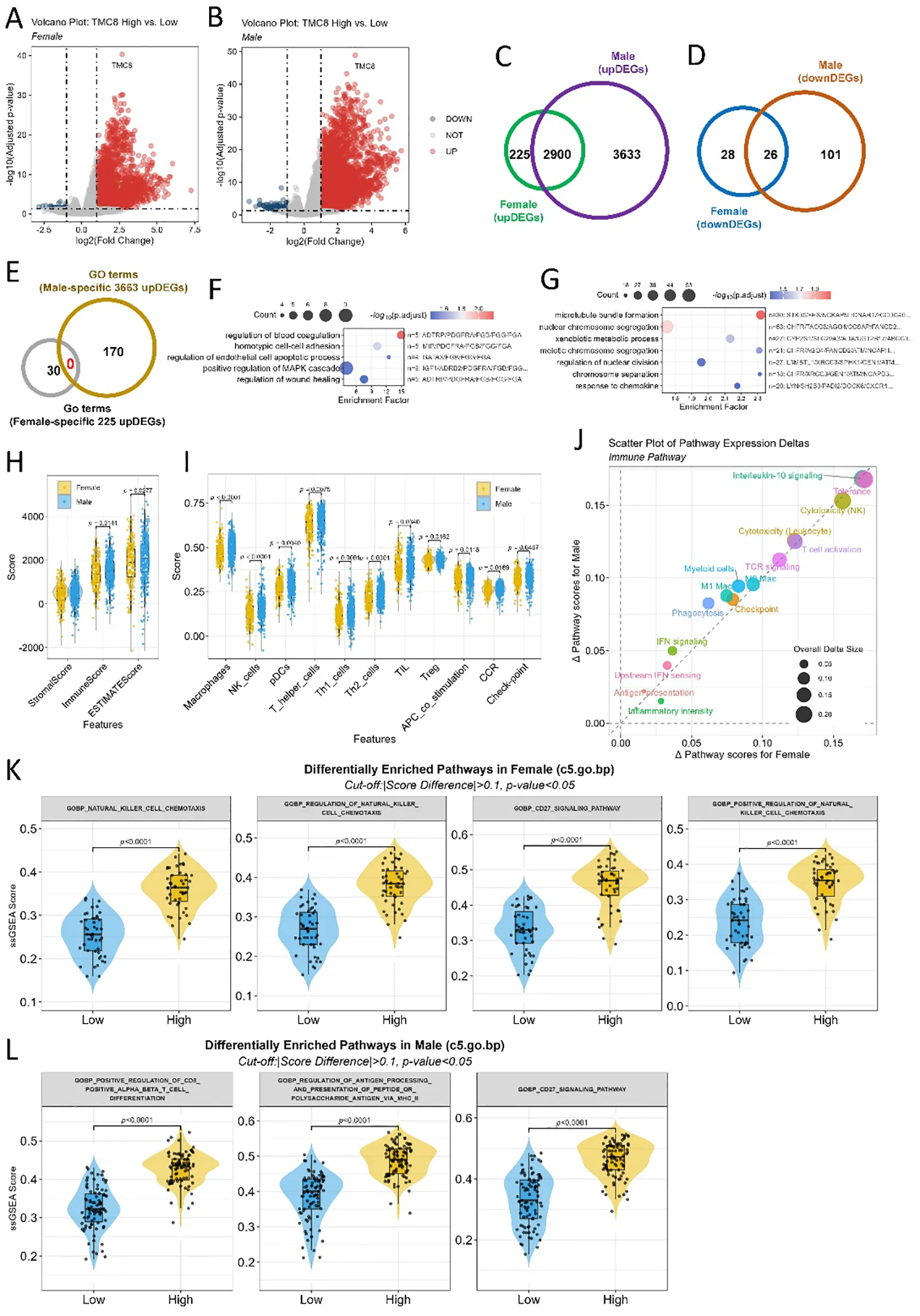
*TMC8* showed distinct transcriptomic and pathway profiles in male ccRCC patients. **(A, B)** Transcriptomic difference analyses between the two *TMC8* expression subgroups across female **(A)** and male **(B)** patients. **(C, D)** Identification of male- and female-specific DEGs. **(E)** Identification of enriched GO terms characterized by the specific DEGs. **(F, G)** Significantly enriched GO biological processes by the specific DEGs in the female **(F)** and male **(G)** patients. **(H, I)** Difference analysis in TME scores and proportion of TILs between the ccRCC individuals with high and low TMC8 expression. **(J)** Scatter plot comparing the magnitude of changes (△pathway scores) in predefined ImmPort pathways between the male and female patients. **(K, L)** Comparison of single-sample gene set enrichment analysis (ssGSEA) on GO biological processes between the two TMC8 expression subgroups across female **(K)** and male **(L)** ccRCC patients.

Further TME characterization revealed that the infiltration proportions of macrophages, Tregs, and immune checkpoint expression were remarkably amplified in male patients versus the females (**Figures 5H, I**). Crucially, larger between-group differences in several immune-related signature scores were observed in males, such as M1 macrophage polarization, IFN signaling, myeloid cells, phagocytosis, checkpoints and antigen presentation (**Figure 5J**). Subsequent ssGSEA based on GO biological processes supported the distinct immunological trajectories, as *TMC8* expression strongly correlated with CD8+ T cell differentiation and antigen presentation in males, whereas the association observed in females involved the regulation of natural killer (NK) cell chemotaxis in females (**Figures 5K, L**). Collectively, these findings reveal a sex-stratified pattern for TMC8, which warrants formal interaction testing.

### 3.7 TMC8 knockdown inhibited ccRCC progression

In addition to its immune-related associations, the significant association between *TMC8* and poor survival suggested it might directly promote ccRCC malignancy. To test this, we evaluated *TMC8* expression in ccRCC cell lines and found *TMC8* was significantly upregulated in cancer cells (Caki-2, A498, and 786-O) versus normal HK-2 cells (**Figures 6A, B**). We then established stable *TMC8* knockdown in A498 and 786-O lines, confirming robust transcript and protein depletion for subsequent functional assays (**Figures 6C–F**). *In vitro*, silencing *TMC8* could attenuate cell viability (**Supplementary Figures 7A, B**) and inhibit clonogenic proliferation (**Figures 6G, H**), which was accompanied by a robust G1/S cell cycle arrest (**Supplementary Figures 7C, D**). Additionally, *TMC8* knockdown markedly suppressed tumor cell migration and invasion, as evidenced by wound healing and transwell assays (**Figures 6I, J**; **Supplementary Figures 7E, F**). *In vivo*, targeted knockdown of *TMC8* significantly suppressed ccRCC progression, resulting in a dramatic reduction in both tumor volume and overall tumor burden compared to control cohorts (**Figures 6K–N**), supporting a role for TMC8 in tumor growth in the xenograft model.

**Figure 6:**
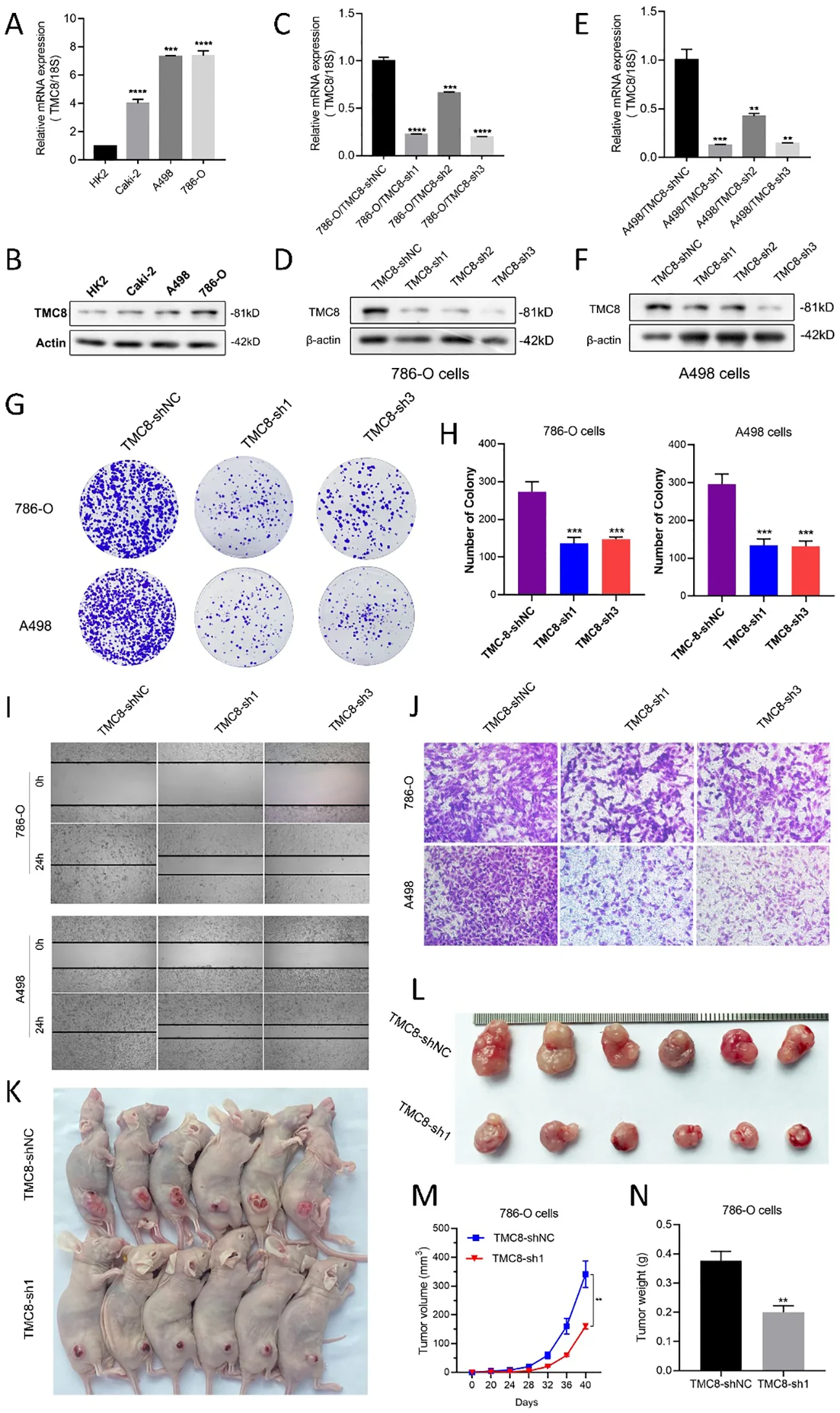
Effects of *TMC8* knockdown on ccRCC cell phenotypes and xenograft tumor growth. **(A, B)** Elevated *TMC8* mRNA and TMC8 protein expression levels in ccRCC cell lines (Caki-2, A498, and 786-O), as assessed by qRT-PCR **(A)** and western blotting **(B)**. **(C–F)** Validation of *TMC8* knockdown efficiency in 786-O **(C, D)** and A498 **(E, F)** cell lines via qRT-PCR **(C, E)** and western blotting **(D, F)**. **(G, H)** Colony formation assays showing that *TMC8* knockdown suppresses cell proliferation. **(I, J)** Wound healing **(I)** and Transwell migration **(J)** assays showing that *TMC8* knockdown was associated with impaired cell migration. **(K–N)** Xenograft models *in vivo* indicating that *TMC8* knockdown significantly reduced tumor growth. **p<0.01, ***p<0.001, ****p<0.0001.

## 4 Discussion

Our multi-platform analyses showed that *TMC8* was coupled to a substantial fraction of the immune transcriptome (28.65%, 406 of 1, 417 immune genes) and overlapped with 41.83% of the prognostic immune-associated genes, suggesting an important role in ccRCC immunity. It’s well known that *TMC8* is a critical determinant of susceptibility to human papillomavirus (HPV) infections, particularly the beta-genus. Typical Epidermodysplasia Verruciformis (EV) results from biallelic mutations in *TMC8*, which stabilizes a heterotrimeric complex with TMC6 and CIB1 in the endoplasmic reticulum by preventing proteasomal degradation (6, 10, 11). Although historically viewed as a keratinocyte-intrinsic restriction factor, the high expression of *TMC8* in lymphoid tissues and its impact on T-cell function suggest that the EV phenotype primarily arises from a subtle cellular immune deficiency (11, 12). In murine models, *TMC8* deficiency leads to reduced CD8+ T cell proliferation and elevated regulatory T cell (Treg) levels, directly impairing the clearance of papillomaviruses (13). Furthermore, head and neck squamous cell cancer (HNSC) also suggested that infiltration proportions of CD4+ and CD8+ T cells were positively associated with *TMC8* expression, supporting an association between *TMC8* expression and T-cell infiltration (12, 14). Importantly, direct evidence for *TMC8* functions in ccRCC has been lacking, while our findings identify *TMC8* expression as associated with multiple immune-related features of the ccRCC TME.

Two previous observations define the conceptual gap addressed here. First, TMC6/TMC8 did not restrict beta-HPV8 gene expression in keratinocytes, suggesting that the EV phenotype may instead reflect an immune cell–intrinsic deficiency (6), a possibility not yet examined at cell-type resolution. Second, a family-wide bulk survey of the TMC family in ccRCC linked *TMC8* expression to prognosis and immune infiltration, but could not identify the compartment carrying the signal and included no functional perturbation (8). Here, by combining single-cell profiling with loss-of-function experiments, we attribute the oncogenic effect of *TMC8* to the tumor cells themselves, resolve a concurrent immune cell–intrinsic program within the same lineage, and provide the first functional evidence for TMC8 in ccRCC.

Our analysis identified *BATF*, *NCF4*, *NOD2*, *ZAP70*, and *ZBP1* as genes positively correlated with TMC8 expression; previous studies have implicated these genes in immune signaling and tumor-related processes. *BATF* acts as a pivotal oncogenic regulator promoting tumor proliferation, epithelial-mesenchymal transition (EMT), and immune evasion across multiple cancers (15, 16). It epigenetically orchestrates Treg differentiation and activation in TME while driving T-cell exhaustion via STAT1/PD-L1 or BATF2/RGS2 axes, making it a high-priority target for combination immunotherapy (15, 17). Conversely, *NCF4* functions as a metabolic and inflammatory sensor that balances reactive oxygen species (ROS) production and inflammasome activation to maintain immune surveillance (18, 19). While its deficiency impairs the IL-18/IFN-γ axis and promotes colorectal tumorigenesis, its identification as a KIRC-specific neoantigen highlights its potential in mRNA vaccine development (20, 21). The intracellular pattern recognition receptor *NOD2* exhibits context-dependent roles, promoting glioblastoma and RCC progression through EMT and cytokine secretion (e.g., IL-8), yet it can also be therapeutically targeted to induce autophagy and apoptosis in colorectal cancer cells (22–24). Similarly, *ZAP70* is indispensable for TCR signaling and anti-tumor efficacy in CD8+ T cells and chimeric antigen receptor (CAR)-NK cells, although its activity is frequently suppressed in TME by metabolic barriers such as cancer-cell-derived fumarate (25–27). Finally, *ZBP1* serves as a critical trigger for anti-tumor necroptosis and PANoptosis by sensing endogenous Z-nucleic acids induced by oxidative stress or pharmacological agents like Kanglexin (28, 29).

A notable feature of *TMC8*-high ccRCC tumors is the concurrent presence of robust immune infiltration and poor clinical outcomes. Despite the increased infiltration of CD8^+^ and CD4^+^ T cells and enrichment of cytotoxic and antigen-processing pathways, this immune-active phenotype does not necessarily translate into effective anti-tumor immunity. One likely explanation lies in the presence of potent immunosuppressive circuits within TME. Tumor-associated macrophages (TAMs), particularly those with an M2-like phenotype, have been shown to suppress CD8^+^ T-cell effector functions through cytokines such as IL-10 and TGF-β and to promote dysfunctional T-cell states in multiple solid tumors (30, 31). Concurrently, chronic antigen exposure in tumors drives sustained expression of inhibitory receptors including *PDCD1* (*PD1*), *HAVCR2* (*TIM3*), and *LAG3* (**Figure 3K**), which defines a transcriptionally and epigenetically stable exhausted T-cell state characterized by reduced cytokine production and cytotoxicity (32, 33). Importantly, previous studies have demonstrated that immune checkpoint expression is enriched in T-cell clusters coexisting with suppressive myeloid populations, and that these interactions promote the shift from effector to exhausted phenotypes even in highly infiltrated tumors (34, 35). Together, these findings suggest a model in which apparent “immune activation” reflects immune cell abundance rather than functionality, while checkpoint upregulation and M2-polarized macrophages establish a dominant suppressive network that ultimately constrains CD8^+^ T-cell effector activity and promotes functional exhaustion.

Across multiple tumor types, male patients generally exhibit higher baseline inflammatory tone but reduced effective anti-tumor immune control, partly due to androgen-driven suppression of adaptive immunity and enhanced myeloid polarization, whereas estrogen signaling in females tends to support more regulated antigen presentation and immune homeostasis (36, 37). In TME, sex hormones modulate macrophage polarization, with androgen signaling favoring immunosuppressive, M2-like phenotypes and estrogen promoting more balanced or tissue-repair–oriented immune responses depending on context (38). Consistently, sex-biased transcriptional programs have been reported in immune cells, including differential interferon responsiveness and antigen presentation capacity, which may amplify divergent responses to identical tumor-intrinsic signals (39). Moreover, exhaustion of CD8^+^ T cells and checkpoint upregulation are more pronounced in immunologically “inflamed yet dysfunctional” male tumor settings, driven by chronic antigen stimulation and suppressive myeloid enrichment (40, 41). Together, these established mechanisms may provide a plausible framework for the pattern observed here, in which TMC8-high tumors in males were associated with stronger immune infiltration but concurrent checkpoint activation and myeloid skewing, consistent with ineffective anti-tumor immunity, whereas females exhibited more restrained immune activation coupled with tissue-maintenance programs.

Beyond its influence on the TME, our *in vitro* and *in vivo* data established *TMC8* as a tumor-intrinsic driver of ccRCC progression, as its knockdown induced G1/S arrest and impaired migratory capacity. This transition from bioinformatic profiling to functional validation supports a dual role for *TMC8* in both the immune microenvironment and intracellular oncogenic programs. Several constraints must be acknowledged.

Firstly, the scRNA-seq annotation lacked malignant epithelial cells, a recognized technical challenge of droplet-based platforms in ccRCC, reflecting preferential loss of fragile, lipid-laden tumor cells during tissue dissociation. Direct tumor-immune crosstalk therefore remains inferred rather than resolved at single-cell resolution; accordingly, the tumor-intrinsic role of *TMC8* rests on direct perturbation in ccRCC cell lines and xenograft assays, whereas the single-cell data characterize the immune compartment. In addition, the single-cell analysis relies on a single dataset, and independent validation is warranted.

Secondly, the immune-related conclusions are primarily correlative rather than causal, and the precise molecular mechanisms remain unclear. Several statistical constraints also apply. The ESTIMATE-based scores partly reflect tumor purity and may be confounded by clinical stage. Correction for multiple testing was applied only to the differential expression analyses. Although multivariable Cox regression was performed to account for relevant clinicopathological variables, residual confounding cannot be excluded. The nominated upstream transcription factors remain computational predictions without experimental validation.

Thirdly, the study lacks dedicated functional assays, and the *in vivo* experiments used immunodeficient mouse models, which preclude evaluation of immune-mediated effects. Thus, functional validation is limited to the tumor-cell phenotypes examined. Immune phenotyping in immunocompetent models is the focus of our ongoing follow-up work.

Finally, although a formal TMC8-by-sex interaction analysis was performed for OS, the sex-stratified immune-related findings remain exploratory and lack functional validation. Future studies, including rescue experiments, mechanistic dissection of the downstream signaling, and orthotopic or metastatic models with survival endpoints, will be essential to substantiate these observations and clarify the multi-layered regulatory roles of *TMC8*.

## 5 Conclusion

Our multi-platform analyses suggest that *TMC8* is associated with a paradoxical TME in ccRCC characterized by concurrent immune activation- and exhaustion-related transcriptional signatures. Despite comparable expression between sexes, its prognostic impact and immunological associations appear markedly stronger in males, consistent with a sex-stratified pattern. Moreover, transcriptomic analyses linked *TMC8* expression to cancer-associated pathway signatures, while knockdown experiments supported its involvement in malignant cell phenotypes. Notably, a causal relationship between *TMC8* and the observed immune-related transcriptional features, particularly whether it directly drives T-cell exhaustion or myeloid immunosuppression, remains to be established through dedicated functional immune assays, toward which our ongoing studies are directed. Collectively, these findings are hypothesis-generating, and *TMC8* represents a putative therapeutic target in ccRCC that warrants further mechanistic characterization.

## Data Availability

The original contributions presented in the study are included in the article/**Supplementary Material**. Further inquiries can be directed to the corresponding authors.
